# TRPC3 Channels in Cardiac Fibrosis

**DOI:** 10.3389/fcvm.2017.00056

**Published:** 2017-09-07

**Authors:** Takuro Numaga-Tomita, Sayaka Oda, Tsukasa Shimauchi, Akiyuki Nishimura, Supachoke Mangmool, Motohiro Nishida

**Affiliations:** ^1^Division of Cardiocirculatory Signaling, Okazaki Institute for Integrative Bioscience, National Institute for Physiological Sciences, National Institutes of Natural Sciences, Okazaki, Japan; ^2^Department of Physiological Sciences, Graduate University for Advanced Studies (SOKENDAI), Okazaki, Japan; ^3^Department of Translational Pharmaceutical Sciences, Graduate School of Pharmaceutical Sciences, Kyushu University, Fukuoka, Japan; ^4^Faculty of Pharmacy, Department of Pharmacology, Mahidol University, Bangkok, Thailand; ^5^Precursory Research for Embryonic Science and Technology, Japan Science and Technology Agency, Kawaguchi, Japan

**Keywords:** Ca^2+^, canonical transient receptor potential, reactive oxygen species, NADPH oxidase, cardiac remodeling, cardiac fibrosis

## Abstract

Cardiac stiffness, caused by interstitial fibrosis due to deposition of extracellular matrix proteins, is thought as a major clinical outcome of heart failure with preserved ejection fraction (HFpEF). Canonical transient receptor potential (TRPC) subfamily proteins are components of Ca^2+^-permeable non-selective cation channels activated by receptor stimulation and mechanical stress, and have been attracted attention as a key mediator of maladaptive cardiac remodeling. How TRPC-mediated local Ca^2+^ influx encodes a specific signal to induce maladaptive cardiac remodeling has been long obscure, but our recent studies suggest a pathophysiological significance of channel activity-independent function of TRPC proteins for amplifying redox signaling in heart. This review introduces the current understanding of the physiological and pathophysiological roles of TRPCs, especially focuses on the role of TRPC3 as a positive regulator of reactive oxygen species (PRROS) in heart. We have revealed that TRPC3 stabilizes NADPH oxidase 2 (Nox2), a membrane-bound reactive oxygen species (ROS)-generating enzyme, by forming stable protein complex with Nox2, which leads to amplification of mechanical stress-induced ROS signaling in cardiomyocytes, resulting in induction of fibrotic responses in cardiomyocytes and cardiac fibroblasts. Thus, the TRPC3 function as PRROS will offer a new therapeutic strategy for the prevention or treatment of HFpEF.

## Introduction

The physiological and pathophysiological significance of Ca^2+^ influx across the plasma membrane in cardiomyocytes has been discussed for a long time, but how the heart decodes a specific Ca^2+^ influx as pathological signal under the background of rhythmic Ca^2+^ entry is obscure. There are two major roles of Ca^2+^ influx in cardiomyocytes: one is to mediate “excitation–contraction (E–C) coupling,” where a local Ca^2+^ influx through voltage-dependent L-type Ca^2+^ channels activated by membrane depolarization (i.e., excitation) induces substantial Ca^2+^ release from sarcoplasmic reticulum (SR), which leads to rhythmic myocardial contraction by increasing myosin ATPase activity through Ca^2+^/troponin C-dependent structural changes of actin-tropomyosin filaments, and the other is to mediate “excitation–transcription (E–T) coupling,” where a local Ca^2+^ influx evoked by neurohumoral excitation and/or hemodynamic load through activation of voltage-independent (or mechano-activated) cation channels induces hypertrophic gene expressions through activating Ca^2+^-dependent transcriptional factors, such as nuclear factor of activated T cells (NFAT) and myocyte enhancer factor (MEF). Transient receptor potential (TRP) proteins, especially canonical TRP subfamily [canonical transient receptor potential (TRPC)] members, have been suggested to function as receptor-activated cation channels (RACCs) regulating E–T coupling in the heart (1). We have also reported that diacylglycerol-activated TRPC3 and TRPC6 heteromultimer channels (TRPC3/6) act as a key mediator of pathological hypertrophy in receptor-stimulated rat cardiomyocytes (2, 3) and pressure-overloaded mouse hearts (4), while our recent studies using TRPC3/6-deficient mice have revealed that TRPC3 specifically mediates pressure overload-induced maladaptive cardiac fibrosis, independently of TRPC6 channels (5, 6). This review focuses on the putative molecular mechanism underlying TRPC3-mediated maladaptive cardiac fibrosis in rodent hearts and discusses its therapeutic possibilities.

## TRPC Channels and Their Physiological Functions

The *trp* gene was first identified in 1989 as a causative gene mutant of phototransduction in *Drosophila* (7). Twenty-eight mammalian TRP homologs have been identified and these are subdivided into six related protein subfamilies based on their genetic and functional similarities: TRPC (canonical), TRPV (vanilloid), TRPM (melastatin), TRPP (polycystin), TRPML (mucolipin), TRPA (ankyrin). TRP proteins commonly possess structural 6 transmembrane domains and preserved 25 amino acid sequence called “TRP domain.” The TRPC family proteins, composed of seven mammalian homologs (TRPC1–TRPC7), are believed as molecular candidates of RACCs (8). TRPC4 and TRPC5 share an about 65% amino acid homology in their group, while TRPC3, TRPC6, and TRPC7 show the best homology covering ~75% of amino acid sequence (9). TRPC1 shares lower sequence homology compared to other TRPC members. TRPC1 is first suggested as a candidate subunit of store-operated Ca^2+^ channels (SOCCs) (10–13). TRPC1 contributes to coordination with elementary Ca^2+^ signaling events though promoting functional coupling between the endoplasmic reticulum (ER) and the plasma membrane in receptor-induced Ca^2+^ signaling (14). TRPC1 also functions as stretch-activated cation channels in mammalian cells (15). Thus, TRPC proteins have two important roles: one is to act as a critical component of stretch-activated or store-operated Ca^2+^ (SOC)-permeable channels and the other is to act as a signaling platform to amplify receptor-activated Ca^2+^ signaling *via* interacting with intracellular signaling molecules (16).

TRPC are generally known to be activated downstream of phospholipase C (PLC)-coupled receptors, such as G-protein-coupled receptors (GPCRs) and receptor tyrosine kinases (16). TRPC proteins comprise non-selective cation channels by forming homo- or hetero-tetramer complex. Due to their universal activation mechanism in many cell types, TRPC channels play important roles in basic cellular responses, including proliferation, differentiation, and death in response to various environmental stimuli. TRPC channels are also linked to physical stimulations such as mechanical stretch, hypoxia, and oxidative stress (17). TRPC1 and TRPC6 are suggested as a component of the tarantula toxin-sensitive mechanosensitive cation channel (15, 18). In fact, inhibition or deletion of TRPC6 has been reported to blunt the chronic mechanical stress-induced muscular contraction in mouse myocytes with Duchenne muscular dystrophy (19). In addition, intracellular lipid mediators, such as diacylglycerol and 20-HETE, also mediate activation of TRPC6 induced by oxidative stress (20) and mechanical stretch (21). Considering the role of TRPC3/6 heterotetramer channels in cardiac hypertrophy, TRPC6 protein signaling complex, including TRPC1 and TRPC3, may function as mechano-activated cation channels in the cardiovascular system.

## Regulation of DAG-Activated TRPC3/C6 Channel Activities

TRPC3/C6/C7 subfamilies are directly activated by diacylglycerol (22, 23). TRPC3 and TRPC6 are mainly expressed in central nervous system, but the physiological significances of both channels have been emerged from vascular physiology. TRPC6 channel is activated downstream of α-adrenergic receptor and mediates cation influx, which evokes membrane depolarization and activation of voltage-dependent Ca^2+^ channel to induce smooth muscle contraction in rat portal vein (24). Following this prominent work, other researches including ours demonstrated that TRPC3/C6 channels function to depolarize the plasma membrane in response to vasoconstrictive GPCR agonists (25, 26). In addition, there are several reports demonstrated the physiological importance of these channels in non-excitable cells. In these cellular contexts, TRPC channels mainly function as Ca^2+^ influx channels. However, because the number and conductance of endogenously expressed TRPC channels seem to be very small, TRPC-mediated Ca^2+^ influx is considered to be involved in local Ca^2+^ signaling rather than global intracellular Ca^2+^ mobilization. In fact, TRPC3-mediated local Ca^2+^ influx is specifically and efficiently transduced to downstream signaling pathways in B lymphocytes (27, 28). TRPC3 is found to interact with several signaling molecules, such as PLC, protein kinase C (PKC), receptor for activated C-kinase-1, inositol 1,4,5-trisphosphate receptor, and calmodulin (27–31). These interactions may be critical for the diversity of downstream signaling pathways induced by TRPC3-mediated local Ca^2+^ influx, since local Ca^2+^ *per se* is highly mobile and easily buffered by buffering proteins in the cytosol.

TRPC3/6 channel activities are negatively regulated by Ser/Thr phosphorylation of TRPC3/6 proteins *via* PKC, protein kinase A (PKA), and protein kinase G (PKG). PKG is reported to phosphorylate human TRPC3 at Thr-11 and Ser-263, and human TRPC6 at Thr-70 and Ser-322 (32). Nitric oxide (NO), atrial natriuretic peptide, and inhibition of phosphodiesterase 5 can activate PKG. The PKG-dependent negative regulation of TRPC6 channel activity by NO is physiologically important in endothelium-dependent vasodilation (33). PKA and PKG recognize a similar substrate sequence, and PKA-dependent phosphorylation of rodent TRPC6 at Thr-69 is found to participate in endothelium-independent vasodilation (26). Increased PKG activity is also reported to suppress Ca^2+^/calcineurin-dependent cardiac hypertrophy induced by agonist stimulation and pressure overload, and blockade of PKG phosphorylation by TRPC6 mutagenesis canceled the PKG-dependent anti-hypertrophic action (34). By contrast, reduction of cGMP/PKG signaling by guanylate cyclase-A gene deletion is reported to develop spontaneous cardiac hypertrophy through TRPC3/6 channel activation (35). In fact, this hypertrophic phenotype was attenuated by the treatment with pyrazole-2, an inhibitor of TRPC1-7 channels.

## TRPC3/6 Channels in Cardiac Remodeling

The heart can adapt itself to various environmental stresses by flexibly changing its structure and morphology. Physiological stimuli, such as physical exercise or pregnancy, induce cardiac hypertrophy to adapt the increases of oxygen and nutrition demands, which is fully reversible. By contrast, pathological conditions also induce cardiac hypertrophy, which is followed by interstitial fibrosis and eventual left ventricular dilation and dysfunction (36). These physiological and pathological cardiac remodelings are chronic tissue responses accompanied with gene expression. Several pieces of evidence indicate the involvement of TRPC channels in the cardiac remodeling processes. Intracellular Ca^2+^ increase and subsequent NFAT activation are the best known pathway that mediates pathological cardiac hypertrophy (37). Thus, TRPC channels were identified as Ca^2+^ permeable channels to activate calcineurin/NFAT pathway. However, now TRPC channels are thought to be not only a cation channel but also a scaffold or membrane anchor to organize downstream signaling complex and participate in pathological cardiac remodeling (Tables 1 and 2). Recently, we have revealed that TRPC3 channel functions as a mediator linking Ca^2+^ signaling and reactive oxygen species (ROS) production which exacerbates pathological cardiac remodeling (5, 6).

**Table 1:** Involvement of TRPC channels in cardiomyopathy.

Gene	Species	Model	Expression and/or function	Reference
TRPC1	Human	Failing heart	Increased expression of mRNA	(38)
	Mouse	Univentricular pressure overload	Increased expression of mRNA	
	Mouse	Pressure overload	Contributed to background Ca^2+^ entry and hypertrophy and fibrosis	(39)
	Rat	Spontaneous hypertensive	Increased mRNA expression and involved in LV	(40)
		rat	hypertrophy	
	Mouse	MI	Increased expression of mRNA	(41)
	Rat	Abdominal aortic banding	Increased protein abundance	(42)
	Rat	Neonatal cardiomyocytes	Knockdown inhibits agonist-induced hypertrophic responses	
	Mouse	Aged mdx mouse	Increased protein abundance	(43)
	Mouse	Dominant negative NRSF transgene	Increased protein abundance	(44)

TRPC3	Mouse	Pressure overload	TRPC3-knockout suppressed cardiac fibrosis and accumulation of oxidative stress	(5, 6)
	Human	Failing heart	Increased expression of mRNA	(38)
	Mouse	Overexpression and chronic agonist treatment	Coupled to NCX1 and involved in arrhythmia	(45)
	Mouse	Cardiac CA-Gα_q_-transgene	Increased expression and involved in hypertrophy and arrhythmia	(46, 47)
	Mouse	MI	Increased expression of mRNA	(41)
	Mouse	Pressure overload	Double knockout with TRPC6 suppressed cardiac remodeling	(19)
	Dog	Tachypacing-induced heart	Increased protein abundance and reduction of atrial	(48)
		failure	remodeling by Pyr3 treatment	
	Human	Atrial fibrillation patient	Increased protein abundance	
	Goat	Atrial fibrillation model by repetitive burst pacing	Increased protein abundance	
	Mouse	Dilated cardiomyopathy (MLP-KO)	Inhibition of TRPC3 suppressed dilated cardiomyopathy and aberrant ROS production	(49)
	Mouse	Pressure overload	Inhibition of TRPC3 suppressed cardiac hypertrophy	(4)
	Rat	Adult cardiomyocytes	Overexpression of TRPC3 increased apoptosis in response to ischemia-reperfusion	(50)
	Rat	Neonatal cardiomyocytes	TRPC3 knockdown reduces PE-induced ANP and BNP expression without affecting cell size and beating frequency	(51)
	Rat	Neonatal cardiomyocytes	Knockdown of TRPC3 suppressed Ang II-induced hypertrophic responses	(3)
	Mouse	Cardiomyocyte-specific transgene	Cardiomyopathy and increased cardiac hypertrophy by pressure-overload and Ang II/PE treatment	(52)
	Rat	Neonatal cardiomyocytes	ET-1, PE, FBS treatment increased the protein abundance	(53)
	Rat	Pressure overload or isoproterenol treatment	Increased protein abundance	
	Mouse	Cardiac CA-calcineurin transgene	Increased protein abundance	
	SHHF rat	Hypertension	Increased protein abundance	

TRPC4	Human	Failing heart	Increased expression of mRNA	(38)
	Mouse	Pressure overload	Contributed to background Ca^2+^ entry and hypertrophy and fibrosis	(39)
	Mouse	MI	Increased expression of mRNA. Ectopic expression of dominant negative TRPC4 increased basal myocyte contractility and reduced hypertrophy and cardiac structural and functional remodeling after MI while increasing survival	(41)

TRPC6	Human	Failing heart	Increased expression of mRNA	(38)
	Mouse	Univentricular pressure overload	Increased expression of mRNA	
	Mouse	Cardiac CA-Gα_q_-transgene	Increased expression and involved in hypertrophy and arrhythmia	(46, 47)
	Mouse	MI	Increased expression of mRNA	(41)
	Mouse	Pressure overload	Double knockout with TRPC3 suppressed cardiac remodeling	(19)
	Mouse	Duchenne muscular dystrophy myocytes	Gene deletion or selective drug blockade of TRPC6 reversed the phenotype of excessive stress-stimulated contractility and arrhythmia	(54)
	Mouse	Isoproterenol stimulation	TRPC6 suppression by Klotho reduced cardiac remodeling	(55)
	Mouse	Pressure overload	Increase protein abundance	(56)
	Mouse	Pressure overload	Phosphorylation of TRPC6 by cGMP-PKG pathway prevented cardiac hypertrophy	(34)
	Mouse	Ang II treatment or TRPC6 overexpression	ANP-induced TRPC6 by phosphorylation protects heart from cardiac hypertrophy	(35)
	Rat	Neonatal cardiomyocytes and cardiac fibroblast	ET-1 treatment increased mRNA and involved in NFAT activation and Gα_12/13_-mediated hypertrophy	(2)
	Human	Failing heart	Increased expression of mRNA	(57)
	Mouse	Pressure overload and endothelin treatment	Increased expression of mRNA	
	Rat	Neonatal cardiomyocytes	Knockdown of TRPC6 suppressed Ang II-induced hypertrophic responses	(3)

TRPC7	Rat	Dahl salt-sensitive rat	Increased expression of mRNA	(58)

*LV, left ventricular; NRSF, neuron-restrictive silencer factor; NCX1, Na^+^/Ca^2+^ exchanger; CA, constitutive active; MLP, muscle LIM protein; PE, phenylephrine; ANP, atrial natriuretic peptide; BNP, brain natriuretic peptide; Ang II, Angiotensin II; ET-1, endothline-1; FBS, fetal bovine serum; SHHF, spontaneously hypertensive heart failure; MI, myocardial infarction; cGMP, cyclic guanosine monophosphate; PKG, protein kinase G; NFAT, nuclear factor of activated T cells; TRPC, canonical transient receptor potential; ROS, reactive oxygen species*.

**Table 2:** Cardiac phenotype of canonical transient receptor potential (TRPC) knockout mice.

Knockout mouse	Phenotype	Reference
TRPC1	No effect on Ang II-induced cardiac hypertrophy	(39)
	Reduced pathological cardiac hypertrophy by double knockout with TRPC4	
TRPC3	Resistant to pressure-overload-induced cardiac remodeling	(4, 5, 18)
	Reduced ischemia–reperfusion (I/R) injury by triple knockout with TRPC6 and TRPC7	(59)
TRPC4	No effect on Ang II-induced cardiac hypertrophy	(39)
	Reduced pathological cardiac hypertrophy by double knockout with TRPC4	
TRPC5	No further reduction of pathological cardiac hypertrophy to that of double knockout of TRPC1/C4	(39)
TRPC6	Resistant to pressure-overload induced cardiac remodeling	(18)
	Reduced I/R injury by triple knockout with TRPC3 and TRPC7	(59)
TRPC7	Reduced I/R injury by triple knockout with TRPC3 and TRPC6	(59)

Canonical transient receptor potential channels were historically presumed to be the molecular entity of SOCCs. Now Stromal interacting molecule 1 and Orai1 channel are identified as a molecular entity of SOCCs. SOC entry is known to be critical for activation of NFAT, which is one of the main transcription factors in cardiac hypertrophy. Therefore, several papers addressed the involvement of TRPC channels in cardiac hypertrophy. Nakayama et al. first demonstrated the involvement of TRPC3 in cardiac hypertrophy (52). It has been known that Ca^2+^ influx and subsequent activation of NFAT play critical roles in cardiac hypertrophy (35, 36). Their group produced transgenic mice overexpressing TRPC3 specifically in cardiomyocytes (37). Those mice showed elevated SOC entry and basal NFAT activity, and eventually exhibited cardiomyopathy. This prominent study clearly indicated that TRPC3 expression *per se* evokes cardiac hypertrophy. Consistent with this report, TRPC3 protein abundance is increased in rodent hypertrophic cardiomyocytes (53). Neurohumoral factor-induced cardiac hypertrophy was also mediated by the increase of TRPC3/C6 expression (3). Ectopic expression of TRPC6 in cardiomyocytes also promoted the induction of pathological cardiac remodeling (57). Consistent with the data obtained from TRPC3/C6 ectopic expression model, cardiomyocyte-specific overexpression of dominant negative mutants of TRPC3 or TRPC6 [N-terminal fragment of TRPC3 or pore-dead mutant (L678-W680 replaced to three alanine residues) of TRPC6] suppressed both neurohumoral factor-induced and pressure-overload-induced cardiac hypertrophy and dysfunction (60). The involvement of TRPC3 was also demonstrated in cardiac remodeling by myocardial infarction (MI) and arrhythmia (41, 48, 50, 61). These reports strongly suggest that TRPC3/C6 channels are prominent molecules mediating cardiac remodeling induced by exposure to several stresses. Recently, we and others reported the effect of TRPC3/C6 genetic deletion on pressure-overload-induced cardiac dysfunction (5, 6, 19). Seo et al. reported that TRPC3/C6 double knockout mice, but not single knockout mice, were resistant to pressure-overload-induced cardiac remodeling (19). However, TRPC3 single deletion was sufficient to suppress cardiac remodeling in response to pressure overload in our study (5, 6). This discrepancy can be partially explained by the difference of mouse strains. In our study, we used 129/Sv mouse and Seo et al. used the mouse backcrossed with C57BL/6 mice. It has been reported that the responses of the heart to pressure overload differ among mouse strains (62). Interestingly, while cardiac hypertrophy was not affected by TRPC3 deletion, cardiac fibrosis was diminished in TRPC3-deficient mice in response to pressure overload (5, 6).

## ROS in Cardiac Physiology and Pathophysiology

**Figure 1:**
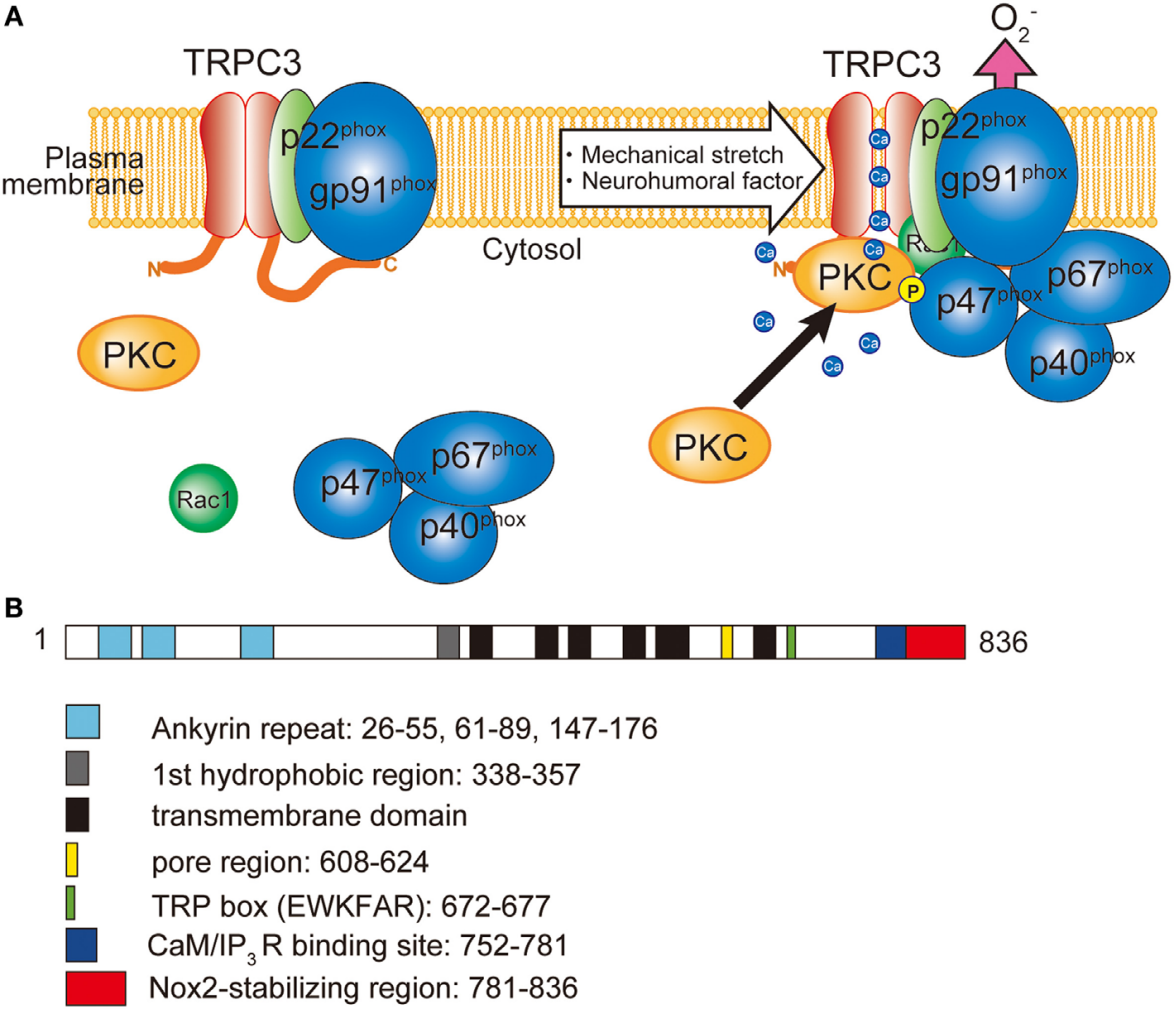
Involvement of TRPC3 in the activation of NADPH oxidase 2 (Nox2). **(A)** TRPC3-mediated Ca^2+^ influx recruits and activates protein kinase C (PKC) which phosphorylates p47^phox^ and evokes Nox2 enzymatic activation. **(B)** Schematic illustration of the domain structure of TRPC3. TRPC3 interacts with Nox2 through the C-terminal region. Numbers represent the positions of amino acids from first methionine.

Production of ROS is observed in most of the pathophysiological conditions of the heart, which exacerbate cardiac remodeling and dysfunction. ROS are generated from both defect of mitochondrial respiratory chains and NADPH oxidase (Nox) activation. Among seven members of Nox proteins, NADPH oxidase 2 (Nox2) and Nox4 are predominantly expressed in the heart. In resting conditions, Nox2 only interacted with p22^phox^ subunit, which is crucial for the expression of Nox2 by preventing proteasomal degradation. Upon cellular activation, other cytoplasmic subunits p67^phox^, p40^phox^, p47^phox^, and small G protein Rac1 are recruited and activate Nox2 protein (Figure 1). Among the cytoplasmic subunits, p47^phox^ mainly regulates Nox2 complex formation. To form complex, phosphorylation of p47^phox^ is necessary. Phosphorylation of p47^phox^ is reported to be mediated by PKC, mitogen-activated protein kinases (MAPKs), and p21-activated kinase (63). Nox2 is located in the membrane of the T-tubules in close apposition to the junctional SR (64). The involvement of Nox in cardiac pathophysiology was demonstrated in myocardial ischemia, pressure-overload and chemical toxicity (65–67). However, Nox plays a critical role in cardiac physiology. During regular heartbeat, diastole is very important regarding intracellular Ca^2+^ homeostasis. Diastolic LV filling causes stretch of cardiomyocytes, which evokes mechano-signal transduction. Prosser et al. demonstrated that mechanical stretch of cardiomyocytes during diastole evokes ROS production *via* Nox2 activation in microtubule-dependent manner (68). Those ROS oxidize ryanodine receptors in junctional SR, which sensitizes ryanodine receptors to Ca^2+^ and thereby increases Ca^2+^ release in coming systolic contraction.

In pathological situations, involvement of cardiac hypertrophy is manifested. Nox activity is increased in the end-stage failing human heart and that it is likely to be an important source of increased cardiac ROS in human chronic heart failure (69). Bendall et al. first reported that Ang II-induced cardiac hypertrophy was blunted by deletion of gp91^phox^ subunit in mice (65). Nox2 mediates Ang II-induced cardiac hypertrophy by modulating Akt and Wnt signaling (70, 71). However, pressure-overload-induced cardiac hypertrophy was not affected by deletion of gp91^phox^ (72, 73). Besides the cardiac hypertrophy, interstitial fibrosis is manifested in heart failure observed in the elderly population, and patients with HFpEF caused by hypertensive heart disease, aortic valve stenosis, and hypertrophic cardiomyopathy (74). Nox2 was important for transforming growth factor β-induced cardiac fibrosis in hypertensive rat (75). gp91^phox^ knockout mice also showed vulnerability to MI. In contrast to the different involvement of cardiac hypertrophy induced by neurohumoral factors versus pressure overload, interstitial fibrosis in response to above factors were abolished in either Nox2 or Rac1-deficient mice (65, 72, 76–78).

Different from tunable Nox2, Nox4 is regulated only by its expression. Nox4 also requires p22^phox^. Therefore, Nox4 is likely to contribute to basal ROS production. It has been demonstrated that Nox4 localizes in intracellular membrane especially perinuclear location associated with SR or mitochondria. Downregulation of Nox4, the major Nox isoform presents during early stages of differentiation, suppressed cardiogenesis. This was rescued by a pulse of low concentrations of hydrogen peroxide (H_2_O_2_) 4 days before spontaneous beating appears. The mechanisms of ROS-dependent signaling included p38 MAPK activation and nuclear translocation of the cardiac transcription factor MEF2C (79). Cardiomyocyte-specific knockout of Nox4 reportedly suppressed pressure-overload-induced cardiac hypertrophy, fibrosis and dysfunction (80). However, null knockout of Nox4 mice showed opposite phenotype as exaggeration of contractile dysfunction, hypertrophy, and cardiac dilatation (81). Cardiomyocyte-specific overexpression of Nox4 counteracted cardiac dysfunction by increasing angiogenic activity in cardiomyocytes, suggesting that increases of Nox4 expression is an adaptive response against chronic heart stress (81). Low tonic production of H_2_O_2_ by Nox4 in endothelial cells has a vasoprotective role by increasing antioxidant systems such as heme oxygenase-1 and NO synthases (82, 83). Therefore, Nox4 seemingly plays a protective role in cardiovascular homeostasis, in contrast to Nox2. Although expression of Nox1 is relatively low in heart compared to Nox2 and Nox4, sepsis-induced myocardial cell death and ROS production were significantly suppressed in Nox1-deficient mice (84).

## Coupling of Nox Proteins and TRPC Channels

**Figure 2:**
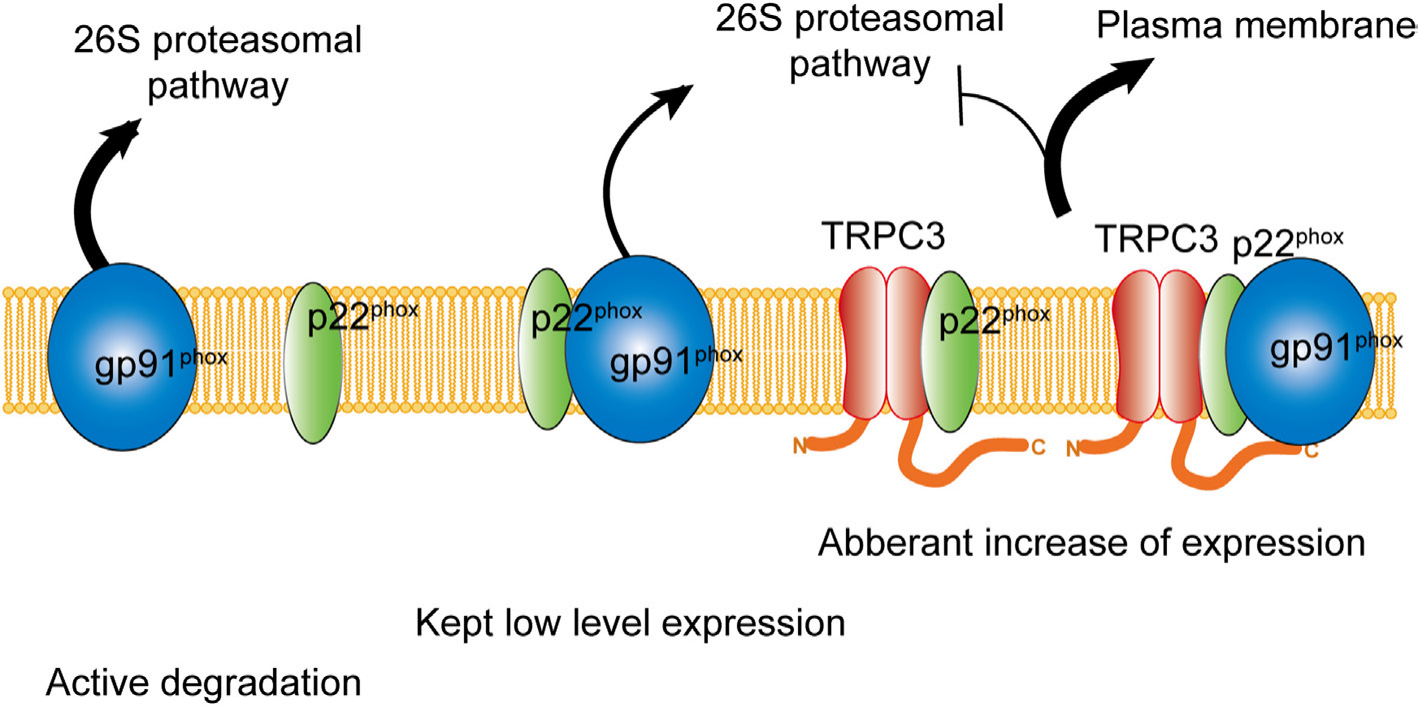
Physical interaction with TRPC3 prevents NADPH oxidase 2 (Nox2) from proteasome-dependent downregulation. In physiological condition, level of Nox2 expression is kept low by proteasomal degradation. Without interaction with p22^phox^, actively gp91^phox^ is degraded. By physical interaction with TRPC3, both gp91^phox^ and p22^phox^ are protected from proteasomal degradation, which leads to excess expression of Nox2 enzyme on the plasma membrane.

Besides the activation mechanism of Nox2 mentioned earlier, Nox2 requires extracellular Ca^2+^ influx to be activated (85–87). In neutrophil-like cell line HL-60, TRPC3, and TRPC6 are critical Ca^2+^ channel for the activation of Nox2 (88). In these cells, GPCR activation induced large increase of intracellular Ca^2+^ concentration and removal or pharmacological blocking attenuated Nox2 activation. Therefore, TRPC channels function as a provider of Ca^2+^ for the enzymatic activation. Kitajima et al. reported that TRPC3 functions not only Ca^2+^ channel but also protein stabilizer by physical interaction (Figures 1 and 2). Previous work demonstrated that interaction with p22^phox^ is critical for Nox2 stabilization. Recently, an ER resident membrane protein competes with p22^phox^ to interact with Nox2. By releasing from p22^phox^ and proceeding to proteasomal degradation, the protein termed negative regulator of ROS facilitates degradation of Nox2 to reduce basal expression (89). By contrast, increased stability of Nox2 by TRPC3 is not simple facilitation of Nox2-p22^phox^ interaction. In fact, p22^phox^ by itself could interact with TRPC3 and be stabilized by the interaction (Figure 2). In pressure-overloaded heart, Nox2 expression was significantly increased, which was completely abolished in TRPC3-deficient mouse hearts. In addition, TRPC3 silencing reduced basal expression of Nox2 in rat neonatal cardiomyocytes (NRCMs), although there was only slight reduction of basal Nox2 expression in normal hearts of TRPC3 knockout mouse compared to those of wild type. In both experimental samples, there were no differences regarding Nox2 mRNA levels. Furthermore, the reduction of Nox2 in TRPC3-silenced NRCMs was mostly rescued by proteasome inhibitor, indicating that TRPC3 increases Nox2 protein abundance by protecting from proteasome-dependent degradation (Figure 2).

In addition, there were reciprocal regulation between TRPC3 and Nox2, i.e., enhancement of Nox2 expression also increased TRPC3 expression and channel function (5). Similar regulation of TRPC channels by Nox protein has been reported. Nox4 expression is important for TRPC6 upregulation in podocytes (90–92). In these studies, TRPC6 was oxidized by ROS produced by Nox4 and its activation was facilitated. However, Nox2-dependent increase of TRPC3-mediated current was not affected by diphenyleneiodonium treatment. Therefore, the reciprocal regulation between TRPC3 and Nox2 also increased channel density on the plasma membrane reflecting the increase of gross expression of TRPC3 by co-expression with Nox2.

Proteomic analysis using RhoA (G17A)-agarose revealed that microtubule-associated Rho guanine nucleotide exchange factor, GEF-H1, was significantly associated with RhoA in TGFβ-stimulated cardiac fibroblasts (6). GEF-H1 is reportedly activated by microtubule depolymerization, and oxidative stress increases GEF-H1 activity through microtubule depolymerization-dependent manner (6). As inhibition of TRPC3 or Nox2 suppressed the mechanical stretch-induced RhoA activation in rat cardiomyocytes and the TGFβ-stimulated RhoA activation in rat cardiac fibroblasts, Nox2-derived ROS-mediated GEF-H1 activation may underlie the induction of fibrotic responses induced by mechanical stress in cardiomyocytes as well as TGFβ stimulation in cardiac fibroblasts.

**Figure 3:**
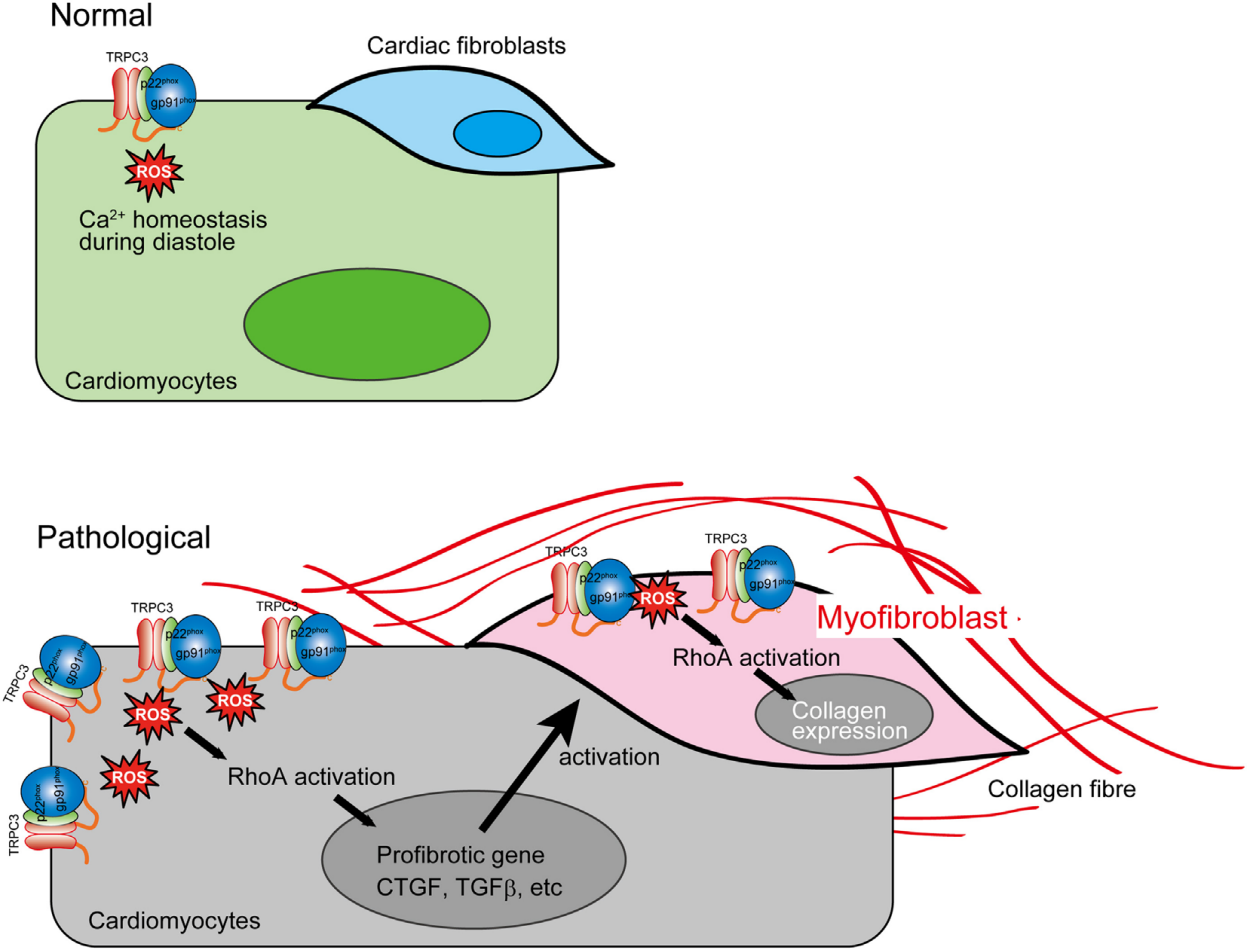
Aberrant reactive oxygen species (ROS) production by TRPC3–NADPH oxidase 2 (Nox2) coupling evokes cardiac fibrosis. In pathological conditions, TRPC3 protein abundance is increased, which leads to Nox2 protein stabilization. This positive regulation of Nox2 induces accumulation of excessive Nox2 complex on the plasma membrane. The ROS production mediated by TRPC3–Nox2 axis activates RhoA in both cardiomyocytes and cardiac fibroblast activated by mechanical stress and TGFβ, respectively, leading to cardiac fibrosis.

The reciprocal positive regulation of TRPC3 and Nox2 caused aberrant increase of ROS production in mechanically stressed hearts, which lead to RhoA activation pathway in both cardiomyocytes and cardiac fibroblasts, resulting in eventual cardiac fibrosis (Figure 3). Interestingly, both TRPC3-deleted and Nox2-deleted mice suppressed only cardiac fibrosis in response to pressure overload (5, 73), while both hypertrophy and fibrosis were reduced in both mice chronically treated with Ang II (2, 63). These pieces of evidence indicate that TRPC3 and Nox2 have close association in pathological cardiac remodeling caused by various environmental stresses.

## Therapeutic Insights

Cardiovascular disease is a leading cause of morbidity and mortality, accounting for more than a quarter of all deaths worldwide (45). Especially, heart failure is a final stage of all cardiovascular diseases, and the 5-year survival rate after diagnosis is less than 50% (93). Since accumulated oxidative stress is the major cause of heart failure, antioxidant agents have been paid attention to the novel therapeutics for heart failure. Based on the involvement of Nox2 in cardiac dysfunction as mentioned above, Nox2-targeted drugs seem to be promising. Several reports demonstrated that inhibitory action on Nox2 ameliorates cardiac dysfunction. Allicin protects against cardiac hypertrophy and fibrosis *via* attenuating ROS-dependent signaling pathways (94). Trimetazidine inhibits pressure overload-induced cardiac fibrosis (95). Nox inhibition ameliorates cardiac dysfunction in rabbits with heart failure by apocynin (96). However, most of Nox inhibitors are less selective among different Nox isoforms. As mentioned above, Nox2 and Nox4 play also critical role in cardiac physiology. Nox is also important for innate immunity. Therefore, complete and direct suppression of Nox enzyme need to be considered with caution. Seo et al. demonstrated that dual inhibitor of TRPC3/C6, GSK503A, could suppress cardiac fibrosis in pressure-overloaded rat hearts (19). In addition, chronic treatment of a relatively selective TRPC3 inhibitor, Pyr3 suppressed mouse cardiomyopathy in either genetic or pressure-overload mouse model of heart failure (4, 49). These reports strongly suggest that TRPC3 could be a potential pharamacological target. Although beneficial effects of Pyr3 on cardiac remodeling was initially caused by suppressing Ca^2+^ influx to activate Nox2 enzymatic activity, chronic treatment of Pyr3 indeed reduced Nox2 protein abundance in cardiomyocytes (5). Since chronic treatment of Pyr3 could interfere the physical interaction between TRPC3 and Nox2, Pyr3 could decrease Nox2 stability by disrupting the interaction with TRPC3. As mentioned above, various environmental stresses increase TRPC3 protein abundance in the heart, which concomitantly amplifies Nox2-mediated ROS production, and eventually evokes pathological cardiac remodeling. Therefore, any intervention that suppresses TRPC3–Nox2 interaction would be a novel therapeutic strategy. Kitajima et al. demonstrated that overexpression of C-terminal fragment of TRPC3 that is a critical region for the interaction with Nox2 in cardiomyocytes abrogated TRPC3 channel activity-dependent ROS production (Figures 1 and 2) (5).

## Conclusion

It will be no doubt that TRPC channels, especially TRPC3, play a key role in the development of maladaptive cardiac remodeling. Although how local background Ca^2+^ entry through TRPC channels specifically encodes signals for induction of hypertrophy has been long discussed, we proposed a new concept of physical interaction-dependent mechanism that TRPC3-mediated local Ca^2+^ influx is directly converted to amplification of Nox2-mediated ROS signaling by stabilizing Nox2 *via* physical interaction between TRPC3 and Nox2. The TRPC3–Nox2 complex-mediated ROS production leads to fibrotic responses in cardiomyocytes and cardiac fibroblasts through activation of ROS-sensitive GEF-H1 (5, 6). These observations will provide a new therapeutic strategy for the prevention and/or treatment of chronic heart failure. On the other hand, more detailed structure-based analyses must be required to understand how TRPC3 specifically stabilizes Nox2 and why closest analog TRPC6 is unable to stabilize Nox2, although the pharmacological significance of TRPC3–Nox2 complex formation through TRPC3 C-terminal region becomes relevant. It is also interesting whether post-translational modification of TRPC3/6 such as Ser/Thr phosphorylation affects the stability of TRPC3–Nox2 complex. These future studies will deepen the understanding of molecular mechanisms underlying regulation of cardiac plasticity by TRPC channels.

## Author Contributions

TN-T, SO, TS, AN, and SM wrote the draft, and MN edited the manuscript.

## Conflict of Interest Statement

The authors declare that the research was conducted in the absence of any commercial or financial relationships that could be construed as a potential conflict of interest.
